# Sweet and Sour *Ehrlichia*: Glycoproteomics and Phosphoproteomics Reveal New Players in *Ehrlichia ruminantium* Physiology and Pathogenesis

**DOI:** 10.3389/fmicb.2019.00450

**Published:** 2019-03-15

**Authors:** Isabel Marcelino, Núria Colomé-Calls, Philippe Holzmuller, Frédérique Lisacek, Yann Reynaud, Francesc Canals, Nathalie Vachiéry

**Affiliations:** ^1^CIRAD, UMR ASTRE, Petit-Bourg, France; ^2^ASTRE, CIRAD, INRA, Université de Montpellier, Montpellier, France; ^3^Unitè TReD-Path (Transmission Rèservoirs et Diversitè des Pathogènes), Institut Pasteur de Guadeloupe, Les Abymes, France; ^4^Proteomics Laboratory, Vall Hebron Institute of Oncology (VHIO), Barcelona, Spain; ^5^CIRAD, UMR ASTRE, Montpellier, France; ^6^Proteome Informatics, Swiss Institute of Bioinformatics, Geneva, Switzerland; ^7^Computer Science Department and Section of Biology, University of Geneva, Geneva, Switzerland

**Keywords:** *Ehrlichia ruminantium*, phosphoproteins, S/T/Y phosphorylation, *N*-glycoproteins, *O*-GlcNAcylated proteins, bacteria physiology, pathogenesis

## Abstract

Unraveling which proteins and post-translational modifications (PTMs) affect bacterial pathogenesis and physiology in diverse environments is a tough challenge. Herein, we used mass spectrometry-based assays to study protein phosphorylation and glycosylation in *Ehrlichia ruminantium* Gardel virulent (ERGvir) and attenuated (ERGatt) variants and, how they can modulate *Ehrlichia* biological processes. The characterization of the S/T/Y phosphoproteome revealed that both strains share the same set of phosphoproteins (*n* = 58), 36% being overexpressed in ERGvir. The percentage of tyrosine phosphorylation is high (23%) and 66% of the identified peptides are multi-phosphorylated. Glycoproteomics revealed a high percentage of glycoproteins (67% in ERGvir) with a subset of glycoproteins being specific to ERGvir (*n* = 64/371) and ERGatt (*n* = 36/343). These glycoproteins are involved in key biological processes such as protein, amino-acid and purine biosynthesis, translation, virulence, DNA repair, and replication. Label-free quantitative analysis revealed over-expression in 31 proteins in ERGvir and 8 in ERGatt. While further PNGase digestion confidently localized 2 and 5 *N*-glycoproteins in ERGvir and ERGatt, respectively, western blotting suggests that many glycoproteins are *O*-GlcNAcylated. Twenty-three proteins were detected in both the phospho- and glycoproteome, for the two variants. This work represents the first comprehensive assessment of PTMs on *Ehrlichia* biology, rising interesting questions regarding ER–host interactions. Phosphoproteome characterization demonstrates an increased versatility of ER phosphoproteins to participate in different mechanisms. The high number of glycoproteins and the lack of glycosyltransferases-coding genes highlight ER dependence on the host and/or vector cellular machinery for its own protein glycosylation. Moreover, these glycoproteins could be crucial to interact and respond to changes in ER environment. PTMs crosstalk between of *O*-GlcNAcylation and phosphorylation could be used as a major cellular signaling mechanism in ER. As little is known about the *Ehrlichia* proteins/proteome and its signaling biology, the results presented herein provide a useful resource for further hypothesis-driven exploration of *Ehrlichia* protein regulation by phosphorylation and glycosylation events. The mass spectrometry proteomics data have been deposited to the ProteomeXchange Consortium with the data set identifier PXD012589.

## Introduction

*Ehrlichia* species are Gram-negative obligate intracellular bacteria, transmitted by ticks. They belong to the *Anaplasmataceae* family in the order *Rickettsiales*. *Anaplasmataceae* includes the genera *Anaplasma*, *Neorickettsia*, and *Wolbachia.* The *Ehrlichia* genus includes *E. chaffeensis* (the causative agent of human monocytic ehrlichiosis, HME) and *E. ewingii* (which causes *E. ewingii* ehrlichiosis). Other species within this genus include *E. canis*, *Panola Mountain Ehrlichia*, *Ehrlichia* muris-like agent (EMLA), and *Ehrlichia ruminantium* (ER), which are predominantly veterinary pathogens but may occasionally infect humans (Maeda et al., 1987; Allsopp et al., 2005; Reeves et al., 2008; Pritt et al., 2011). Three news species of cattle-related *Ehrlichia* spp. (closely related to *E. canis*) have been recently reported (Cabezas-Cruz et al., 2014). At the moment, there is no effective commercial vaccine available for any of the *Ehrlichia* species, with impact in human and veterinary health. Avoidance of tick bites remains the mainstay of prevention (Thomas et al., 2016).

Infection with *Ehrlichia* species in mammalian cells begins with the intracellular uptake of the infectious extracellular form of the organism, the EB or DC. After EB/DC endocytosis, the bacterium replicates and matures to form a RB or RC and then morula before redifferentiating into EB/DC that leaves the infected host cell after lysis to spread infection (McClure et al., 2017). During this process, *Ehrlichia* utilizes many evasion mechanisms including suppression of apoptosis of host cells, modulation of chemokine and cytokine responses, and down-regulation of host pattern recognition receptors that might enable clearance of the infection (Thomas, 2016). *Ehrlichia* life cycle in the tick vector is still not fully elucidated. To adapt and survive to the different growth conditions both in mammalian and arthropod hosts, the bacterium relies on differential gene expression, but also on the modification of proteins and other molecules.

Post-translational modifications are one of the most important mechanisms for activating, changing, or suppressing protein functions, being widely used by pathogens to interact with their hosts (Ribet and Cossart, 2010; Prabakaran et al., 2012; Beltrao et al., 2013; Salomon and Orth, 2013; Cain et al., 2014; Ravikumar et al., 2015; Müller, 2018). Presently, over 450 different PTMs are identified (including phosphorylation, glycosylation, acetylation, succinylation, pupylation, etc.) expanding the diversity of the proteome enormously (Prabakaran et al., 2012; Striebel et al., 2014; Pisithkul et al., 2015; Singhal et al., 2015; Mijakovic et al., 2016; Eichler and Koomey, 2017; Christensen et al., 2018; Gaviard et al., 2018). These PTMs are not genetically encoded and they can have many different consequences for the affected proteins and the cellular processes they are involved in Prabakaran et al. (2012). Phosphorylation is one of the most studied PTMs in bacteria, being related to cell signaling (Mijakovic et al., 2016). Glycosylation is the most abundant and diverse form of modification, impacting protein folding, trafficking, processing, stability, biological activity (Eichler and Koomey, 2017), and bacterial pathogenesis (Poole et al., 2018).

Different types of PTMs have been reported in *Ehrlichia*. Inside the host cells, *E. chaffeensis* immunoreactive tandem repeat proteins (TRPs) TRP47 and TRP75 and *E. canis* TRP95 were shown to be tyrosine phosphorylated, presumably by host tyrosine kinases (Wakeel et al., 2010; McBride and Walker, 2011). Luo et al. (2017) revealed that *E. chaffeensis* TRP120 can exploit host SUMOylation system during the infection process in order to establish its intracellular niche. Other *Ehrlichia* proteins involved in host immune response such as the immunodominant 28 kDa outer membrane proteins (p28-Omp) in *E. chaffeensis* and *E. canis* can be modified by phosphorylation and glycosylation (Ganta et al., 2009). Map-1, the major antigenic protein in ER and homologous to p28-Omp, was also found to be glycosylated (Postigo et al., 2008). Faburay et al. (2017) recently showed that such PTMs can have a significant impact for the development of effective vaccine. Indeed, the production of subunit vaccine based on glycosylated Map-1 recombinant protein was able to induce a specific humoral and Th1 type T-cell responses against ER in sheep, after vaccination (Faburay et al., 2017).

Nevertheless, all of these studies are focused on a limited number of already identified proteins. To understand the link between protein phosphorylation/glycosylation and bacterial adaptation and survival, it is important to have a global view of the PTMs events taking place at a given time, under specific conditions. In 2015, our group showed on ER EBs from both virulent and attenuated variants, that 85% of the identified proteins were present in more than one proteoform, suggesting that PTMs are important in ER biology (Marcelino et al., 2015). Bioinformatics analyses revealed that some proteoforms of the most abundant proteins Map-1 and Porin ERGA_CDS_05140 were *N*-glycosylated proteins (Marcelino et al., 2015).

Herein, using mass-spectrometry-based assays, we focused on the most common PTMs – phosphorylation and glycosylation – to explore how PTMs could modulate ER biological processes, including pathogenesis. For this, we characterized the proteome, the S/T/Y phosphoproteome, and the glycoproteome of ER Gardel variants with different levels of virulence *in vivo* and different growth kinetics *in vitro* (Marcelino et al., 2015). Briefly, attenuated ER Gardel variant (ERGatt) induces mild clinical signs (hyperthermia) on naïve goats and strong protective immune response after challenge, whereas *in vitro*, the life cycle of ERGatt is shorter (96 hpi) than for the virulent variant (ERGvir, 120 hpi). For virulent and attenuated variants, cell lysis occurs with the release of infectious EBs *in vitro* (Marcelino et al., 2015). In this work, we used purified extracellular and infectious EBs from virulent and attenuated ER Gardel variants and optimized existing enrichment protocols for glycoproteins [using Ultralink hydrazide (ULH) resin through the oxidation and non-oxidation of sugars moieties] and phosphopeptides [using titanium dioxide (TiO_2_) beads] detection, followed by mass spectrometry analysis for protein identification. Phospho- and glycoproteins differentially detected in both virulent and attenuated variants are also discussed, as these could be key elements in the interactions with the host cell, and therefore represent interesting targets to interfere with the ER infectious process.

## Materials and Methods

### Bacterial Strains

*Ehrlichia ruminantium* Gardel virulent strain (passages 17, 19, and 24) and ERGatt (passages 240, 242, and 244) variants were grown in bovine aortic endothelial cells (BAECs), as described elsewhere (Marcelino et al., 2015). When 80% cell lysis was observed, infectious and extracellular EBs were harvested and then used to (i) infect a new confluent monolayer of BAEC or (ii) to be purified using a multistep centrifugation methodology (Peixoto et al., 2007). Purified EBs were stored at -80°C with a “Complete EDTA-free” anti-protease cocktail (Roche, Germany) and anti-phosphatases (Roche, Germany) prior to proteomic analysis.

### Protein Extraction

Elementary bodies protein extracts were prepared using a 2D-DIGE buffer (7 M urea, 2 M Thio-urea, 4% CHAPS, 30 mM Tris, pH 8.5) and several cycles of sonication on ice (Vibra Cell with Microtip W72403, from Sonics and Materials; settings: 30% duty cycle + 2 microtip limit, pulsed duty cycle 20). After sonication, homogenates were centrifuged at 15,000 × *g* for 10 min at 4°C to remove debris. The total amount of protein per ER sample (three biological replicates per variant) was quantified using the 2DQuant kit (GE Healthcare) according to the manufacturer’s instructions.

### FASP Protocol for Protein Digestion

Twenty micrograms of ER protein extract (solubilized in 2D-DIGE buffer) were loaded in the upper part of a microcon-10 kDa centrifugal unit (Millipore). Proteins were washed three times with 200 μl of 8 M urea in 50 mM ammonium bicarbonate (NH_4_HCO_3_) using a multistep centrifugation process [at 14,000 × g, RT (25°C), 20 min]. Flow-through was discarded after each centrifugation step. Proteins were reduced by adding 100 mM dithiothreitol (DTT), prepared in 8 M urea in 50 mM NH_4_HCO_3_ with 1 h incubation at RT. After three washing steps with 8 M urea in 50 mM NH_4_HCO_3_ (centrifugation at 14,000 × g, 20 min, at RT), the proteins were alkylated by adding 50 mM iodoacetamide (IAA) (prepared in 8 M urea in 50 mM NH_4_HCO_3_) at RT and kept in dark for 30 min. Then, proteins were washed two times with 8 M urea in 50 mM NH_4_HCO_3_ and then three times in 50 mM NH_4_HCO_3_. The upper filter was then transferred to a new collection tube. Trypsin gold (Promega, Madison, WI, United States) was added to the samples at a ratio of protein:trypsin 30:1, and the samples were incubated at 37°C for 18 h in 50 mM NH_4_HCO_3_. Tryptic peptides were then eluted by centrifugation at 14,000 × g, 10 min, at RT. Formic acid (FA) was added at a final concentration of 0.5% and peptides were stored at -20°C until further use.

### Phosphopeptide Enrichment With TiO_2_ Chromatography

The phosphopeptide enrichment was performed according to a protocol developed by Thingholm et al. (2006) with some modifications. Briefly, tryptic peptides (originated from 250 μg of protein extract per biological replicate) were prepared using the FASP protocol described above. Peptides were then diluted in 60% acetonitrile (ACN) with 1% trifluoroacetic acid (TFA). TiO_2_ beads at 0.50 mg/μl were previously equilibrated in 1 M glycolic acid, 80% ACN, and 1% TFA. To allow an effective peptide binding to TiO_2_ beads, 0.6 mg TiO_2_ per 100 μg of peptides was used. Solution was incubated during 20 min at RT, with end-over-end rotation, to avoid loss of specificity. Solution was centrifuged in a bench top centrifuge at max speed (15,600 × *g*, 2 min, RT), and supernatant containing non-phosphorylated peptides was discarded.

TiO_2_ beads with phosphopeptides were loaded on previously prepared in-house constructed stage tips (made using high performance C18 extraction disks into pipette tips). After two successive washes with 60% ACN and 1% TFA, bound phosphopeptides were eluted first with 5% NH_4_OH and then with 10% NH_4_OH with 25% ACN. Eluted phosphopeptides were evaporated, resuspended in 0.1% FA, and stored at -20°C until further analysis.

### Glycoprotein Enrichment With Ultralink Hydrazide Resin

Selective enrichment of glycoproteins was performed using ULH resin according to a protocol developed by Chen et al. (2016), with some modifications. As this protocol is not fully specific for *Ehrlichia* oxidized proteins (and therefore glycosylated proteins), it is necessary to use a non-oxidized protein extracts to identify false positive during the analysis (raw M/MS data available in PRIDE archive ID: PXD012589). Proteins extracts (500 μg per biological replicate) previously prepared in 2D-DIGE buffer were first precipitated using the 2D Clean-up kit (GE Healthcare). Proteins were then solubilized using the coupling buffer NP40 [PBS with 1% Nonidet P-40 (NP40) and 0.1% SDS, pH 7.2], using several cycles of sonication on ice, as described above. The suspension was split in two for oxidative and non-oxidative (control) sample preparation conditions. The control sample was treated like the oxidized sample but without the addition of sodium metaperiodate (NaIO_4_).

For the oxidation of the sugar moieties, 15 mM NaIO_4_ was added to the solution, and proteins were incubated in the dark for 30 min with end-over-end agitation. NaIO_4_ was removed using microcon-10 kDa centrifugal unit (Millipore) and three washing steps with NP40 buffer was made. A brief sonication step on ice was made to reduce some protein aggregation. Afterward, 250 μl of previously prepared ULH resin (Pierce) was added, and the coupling reaction was shaken overnight at RT. After coupling, beads were washed three times with 100 mM Tris–HCl (pH 8) containing 2% sodium dodecyl sulfate (SDS) and once with 50 mM NH_4_HCO_3_. Glycoproteins conjugated to hydrazide beads were denatured by adding 8 M urea in 50 mM NH_4_HCO_3_. Proteins were reduced by adding 100 mM DTT with 1 h incubation at RT with agitation. After three washing steps with 8 M urea in 50 mM NH_4_HCO_3_, the proteins were alkylated by adding 50 mM IAA (prepared in 8 M urea in 50 mM NH_4_HCO_3_) at RT and kept in dark for 30 min, with agitation. Then, beads were washed three times with 50 mM NH_4_HCO_3_. After washing, trypsin (Promega, Madison, WI, United States) was added to the samples at a ratio of protein:trypsin 30:1, and the samples were incubated at 37°C for 18 h in 50 mM NH_4_HCO_3_. The tryptic peptides were collected and desalted with C18 zip-tips (Millipore). The beads were further washed sequentially with 0.5% Triton X100 in 50 mM NH_4_HCO_3_, 5 M NaCl, 100 mM sodium carbonate (Na_2_CO_3_, pH 12), and 50 mM NH_4_HCO_3_ for three times. N-linked glycopeptides were released by adding 2 μl of PNGase F (New England Biolabs, Ipswich, MA, United States) and incubated at 37°C overnight with agitation. Deglycosylated peptides were also collected and desalted with C18 zip-tip.

### nLC–MS/MS Analysis

Peptides (from trypsin or PNGase F digestion) were analyzed using a linear ion trap Velos-Orbitrap mass spectrometer (Thermo Fisher Scientific, Bremen, Germany). Instrument control was performed using Xcalibur software package, version 2.2.0 (Thermo Fisher Scientific, Bremen, Germany). Peptide mixtures were fractionated by on-line nanoflow liquid chromatography using an EASY-nLC system (Proxeon Biosystems, Thermo Fisher Scientific, Bremen, Germany) with a two-linear-column system. Digests were loaded onto a trapping guard column (EASY-column, 2 cm long, ID 100 μm, and packed with Reprosil C18, 5 μm particle size from Proxeon, Thermo Fisher Scientific, Bremen, Germany) at 4 μl/min. Then, samples were eluted from the analytical column (EASY-column, 10 cm long, ID 75 μm, and packed with Reprosil, 3 μm particle size from Proxeon, Thermo Fisher Scientific, Bremen, Germany). Separation was achieved by using a mobile phase from 0.1% FA (Buffer A) and 99.9% ACN with 0.1% FA (Buffer B) and applying a linear gradient from 0 to 35% of buffer B for 60 min for phosphopeptide analysis and from 3 to 35% of Buffer B for 90 min for peptides obtained from the glycoprotein enrichment analysis at a flow rate of 300 nl/min. Ions were generated applying a voltage of 1.9 kV to a stainless steel nano-bore emitter (Proxeon, Thermo Fisher Scientific, Bremen, Germany), connected to the end of the analytical column, on a Proxeon nano-spray flex ion source.

The LTQ Orbitrap Velos mass spectrometer was operated in data-dependent mode. A scan cycle was initiated with a full-scan MS spectrum (from *m*/*z* 300 to 1600) acquired in the Orbitrap with a resolution of 30,000. The 20 most abundant ions were selected for collision-induced dissociation fragmentation in the linear ion trap when their intensity exceeded a minimum threshold of 1000 counts, excluding singly charged ions. Accumulation of ions for both MS and MS/MS scans was performed in the linear ion trap, and the AGC target values were set to 1 × 10^6^ ions for survey MS and 5000 ions for MS/MS experiments. The maximum ion accumulation time was 500 and 200 ms in the MS and MS/MS modes, respectively. The normalized collision energy was set to 35%, and one microscan was acquired per spectrum. Ions subjected to MS/MS with a relative mass window of 10 ppm were excluded from further sequencing for 20 s. For all precursor masses a window of 20 ppm and isolation width of 2 Da were defined. Orbitrap measurements were performed enabling the lock mass option (*m*/*z* 445.120024) for survey scans to improve mass accuracy.

### Data Processing

All raw files were searched against the UniprotKB ER database (Bateman et al., 2017) (downloaded on January 2017 with 4148 entries and updated on December 2017) with MaxQuant version 1.5.5.1 [Max Planck Institute of Biochemistry (Cox and Mann, 2008)]. The parameters were set as follows:

(a) For phosphoproteome: search criteria included a fixed carbamidomethylation of cysteines, and variable modifications of oxidation on methionine residues, and phosphorylation on serine, threonine, or tyrosine residues were searched. Search was performed with trypsin digestion and allowed a maximum of two missed cleavages on the peptides analyzed from the sequence database. The false-discovery rates (FDRs) of proteins, peptides, and phosphosites were set at 0.01. The minimum peptide length was seven amino acids, and a minimum Andromeda score was set at 40 for modified peptides. A site localization probability of 0.75 was used as the cutoff for localization of phosphorylation sites. For advanced identification, the Second Peptide Search (SPS) in MS2 spectra and the Match Between Runs (MBR) feature were enabled.
(b) For whole proteome and glycoproteome: common search criteria included a fixed carbamidomethylation of cysteines and variable modifications of oxidation on methionine residues. Search was performed with trypsin/P digestion and allowed a maximum of two missed cleavages on the peptides analyzed from the sequence database. The FDR from proteins and glycoproteins was set at 0.01. The minimum peptide length was seven amino acids, and a minimum Andromeda score was set at 40 for modified peptides. For advanced identification, the SPS in MS2 spectra and the MBR feature were enabled.
(c) For *N*-glycoproteins: parameters were similar to glycoproteomics (main search peptide tolerance: 4.5 ppm; digestion: trypsin/P, max. 2 missed cleavages; fixed modification: cysteines carbamidomethylation; variable modification in the tryptic peptide fraction: methionine oxidation, FDR set at 0.01, SPS and MBR were enabled). For PNGase F fractions, variable modifications were: methionine oxidation and asparagine deamidation. LFQ of proteins with normalization was done in MaxQuant.

The mass spectrometry proteomics data have been deposited to the ProteomeXchange Consortium^1^ via the PRIDE partner repository (Vizcaíno et al., 2014) with the dataset identifier “PXD012589”.

### Bioinformatics

Known contaminants from MaxQuant database and reverse identified peptides/proteins were discarded. All the statistical analyses of the MaxQuant output tables were performed with the Perseus program (version 1.5.6.0), which is a component of the MaxQuant distribution. The tables were filtered to remove contaminants and reversed sequences. Intensities were log(2) transformed and the missing values of intensities were replaced by normal distribution with a downshift of 1.8 SDs and a width of 0.3 SDs. The differentially expressed proteins between ERGvir and ERGatt were identified by the *P*-value (*P* < 0.05), which is significant based on a two-sample *t*-test with a permutation-based FDR cutoff 0.05 with S0 set on 0.2 for all data sets.

Furthermore, to identify *N*-glycosylated proteins, only modified asparagines (*N*) within the canonical sequence motif of *N*-glycosylation defined by the consensus motif N–X–S/T (X = !P), were accepted as true glycosylation sites. For identification of phosphorylation site motifs, we used the Sequence Logos algorithm in Perseus. Predicted Gene Ontology (GO) cellular functions of identified proteins with high confidence from the two ER variants were obtained from UniprotKB and using Perseus Annotation tools. Whenever an identified protein had an unknown function and/or localization, manual reannotation was performed using several databases such as KEGG, Interproscan, etc.

### Detection of O-Glycosylated Proteins Using 2D Western Blotting Coupled to MALDI–MS/MS

#### 2D Gels Preparation

For protein isoelectric focusing (IEF), 50 μg of proteins (pool of three ERGvir or ERGatt biological replicates described above) was precipitated using 2D clean-up kit (GE Healthcare Bio-sciences, Uppsala, Sweden). The pellet were resolubilized in rehydration buffer (7 M urea, 2 M thio-urea, 4% CHAPS, 50 mM DTT, 0.5% IPG buffer pH 3–10 non-linear, 0.002% bromophenol blue). To ensure a total solubilization of the proteins pellet, sample was incubated at RT for 1 h, followed by homogenization under vigorous agitation (vortex). The sample was applied on a pre-cast immobilized pH gradient (IPG) strips (7 cm, pH 3–10, non-linear, GE Healthcare Bio-sciences, Uppsala, Sweden) and covered with mineral oil. Complete sample uptake was carried out overnight for a passive rehydration at RT. Protein IEF was carried out at 20°C under a current limit of 75 μA per strip on IPGphor3 cell (GE Healthcare Bio-sciences, Uppsala, Sweden) and performed at 100 V for 1 h (Step mode), then 300 V for 2 h (Gradient mode), followed by a ramping to 1000 V for 4 h and to 4000 for 3 h (Gradient mode), and was completed at 4000 V for 3 h (Step mode) for a total of 22,600 Vh (in 13 h).

After IEF, the IPG strips were equilibrated for 15 min at RT under gentle agitation in reducing solution (50 mM Tris–HCl, pH 8.8, 6 M urea, 30% glycerol, 2% SDS, 0.002% bromophenol blue; 2% w/v DTT). They were then equilibrated for a further 15 min in an alkylating solution, which was identical to the reducing solution except that the DTT was replaced by 2.5% (w/v) IAA. The equilibrated IPG strips were applied to the top of a pre-cast acrylamide gel (pre-cast NuPAGE 4–12% Bis–Tris Zoom, Invitrogen) and sealed with a concentrating agarose solution (GE Healthcare Bio-sciences, Uppsala, Sweden) pre-heated to 80°C. Electrophoresis was carried out at 40 V, 400 mA, and 50 W for 1 h and then at 100 V, 400 mA, 50 W for 3 h at RT with the XCell SureLock^TM^ Mini-Cell Electrophoresis System (Invitrogen) in running buffer (MES, Invitrogen). Once electrophoretic separation achieved, gels were rinsed in ultrapure water to remove the excess of SDS. After electrophoresis, gels were used for total protein staining or western blotting as described below.

Total levels of proteins were revealed using Colloidal Coomassie Blue, according to methodology described by Marcelino et al. (2015). Briefly, gels were stained for 24 h in Colloidal Coomassie Blue and subsequently washed three times in double distilled water. Gels were stored at 4°C in a 20% (w/v) ammonium sulfate solution until image acquiring and spot excision. Digital images of the gels were acquired using a laser-based scanner Typhoon FLA 9500 (GE Healthcare Bio-sciences, Uppsala, Sweden).

#### Western Blot for *O*-GlcNAc Glycoprotein Detection

Western blotting was performed using the specific primary antibody anti-*O*-GlcNAc monoclonal (MAb CTD 110.6) (Santa Cruz Biotechnology, United States) as it is known to recognize a wide range of *O*-GlcNAcylated proteins (Ma and Hart, 2014) and because there is no cross-reactivity with the N-linked oligosaccharides.

After 2DE, proteins were transferred to a nitrocellulose membrane, using the XCell II^TM^ Blot Module (Invitrogen) at 30 V for 1 h. After the transfer, the membrane was stained with Ponceau to ensure that the transfer was successful. After several washing steps with milliQ water, the membrane was blocked for 1 h at RT in Blocking buffer (Thermo Fisher), under gentle agitation. Membrane was then incubated overnight at RT with the specific primary MAb CTD 110.6 (diluted 1:200 in Blocking buffer) in a rocking platform. After several washing steps with PBS-Tween 20 (PBS-T, 15-20 ml), the membrane was incubated for 1 h at RT with HRP-conjugated secondary antibody anti-mouse IgM-HRP (Thermo Fisher) (diluted 1:5000 in blocking buffer), in a rocking platform. After three washing steps (10 min) with PBS-T, HRP subtract Peroxide solution and Luminol solution was added at 1:1 ratio (0.12 ml of solution per cm^2^ of the membrane). After 2 min incubation in the dark, the excess of substrate was removed and the membrane was put in the GE imager tray to detect the chemiluminescence in automatic mode. Image capture of nitrocellulose membrane was performed using ImageScanner III (GE Healthcare Bio-sciences, Uppsala, Sweden).

#### Spot Picking and In-Gel Digestion

Spots identified as *O*-GlcNAc glycoproteins by western blot were manually excised from a corresponding 2D gel stained with Coomassie. Spots were excised with a 1000 μl tip and transferred to a 96-well collecting plate containing 120 μl of a 10% ethanol solution. Plates with excised spots were stored at -20°C until further use.

The proteins of interest were digested into their component peptides with trypsin and eluted from the gel plugs, as described previously (Marcelino et al., 2015). Briefly, spots were excised, destained, reduced with DTT, alkylated with IAA, and dried in a speedvac. Gel pieces were rehydrated with digestion buffer (50 mM NH_4_HCO_3_) containing trypsin (6.7 ng/μl) (Promega, Madison, WI, United States) and incubated overnight at 37°C. The tryptic peptides were acidified with FA, desalted, and concentrated using homemade reversed phase microcolumns (POROS R2, Applied Biosystems, Foster City, CA, United States). The peptides were eluted onto a MALDI plate using a matrix solution that contained 5 mg/ml α-cyano-4-hydroxycinnamic acid dissolved in 50% (v/v) ACN/0.1% (v/v) FA.

#### Protein Identification

Protein identification was undertaken on a 4800 MALDI-TOF/TOF instrument (Applied Biosystems, Framingham, MA, United States) with 4000 series explorer v3.5 software in both MS and MS/MS mode. Each MS spectrum was obtained in a result independent acquisition mode with a total of 800 laser shots per spectrum, with internal calibration using Angiotensin II (1046.2 Da), Angiotensin I (1296.5 Da), Neurotensin (1672.9 Da), ACTH [1–17] (2093.5 Da), and ACTH [18–39] (2465.7 Da) (PepMix1, from LaserBio Labs). Fifteen signal-to-noise (S/N) best precursors from each MS spectrum were selected for MS/MS analysis, starting with the most intense peak and ending with the least intense peak. MS/MS analyses were performed using Collision-induced dissociation (CID) assisted with air, using a collision energy of 1 kV and a gas pressure of 1 × 10^-6^ Torr. A total of 1400 laser shots were collected for each MS/MS spectrum with the peak detection minimum S/N setting at 20.

For protein identification in 2D gels, Mascot Generic Files combining MS and MS/MS spectra were automatically created and used to interrogate a non-redundant protein database using a local version of Mascot v2.2 from Matrix Science through the Global Protein Server (GPS) v3.6 (Applied Biosystems, Foster City, CA, United States).

## Results

### Phosphoproteome Characterization

**Figure 1:**
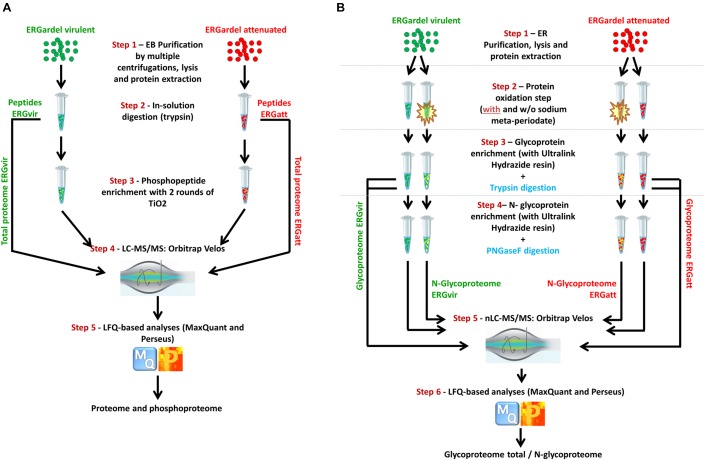
Overall workflow for integrated profiling of the proteomes, phosphoproteomes, glycoproteomes, and *N*-glycoproteomes of ERGvir and ERGatt. Infectious ER EBs (three biological replicates per variant) were harvested at cell lysis, purified, and lysed for protein extraction. For proteome profiling, tryptic digestion was performed **(A)**. Part of the tryptic peptides were used for TiO_2_ enrichment for phosphoproteome profiling **(A)**. For glycoproteome and *N*-glycoproteome profiling **(B)**, protein extracts were oxidized (highlighted in yellow) and incubated with ULH resin (non-oxidized sample was included as control to avoid for false-positive identifications). All data were acquired with nLC–MS/MS and analyzed with MaxQuant and Perseus softwares.

The workflow for the analysis of phosphoproteome of ERGvir and ERGatt is illustrated in Figure 1A. Three biological replicates per ERGvir and ERGatt EBs were harvested at host cell lysis and purified by a multistep centrifugation process as described previously (Step 1). Proteins were extracted and peptides generated using trypsin (Step 2). Phosphopeptides were enriched (Step 3) and analyzed by nLC–MS/MS on a high-speed, high-resolution mass spectrometer (Step 4). Peptide identifications and protein expression analysis were done with MaxQuant followed by Perseus analysis (Step 5). Tryptic peptides were also used for proteome characterization, as described in Figure 1A.

**Figure 2:**
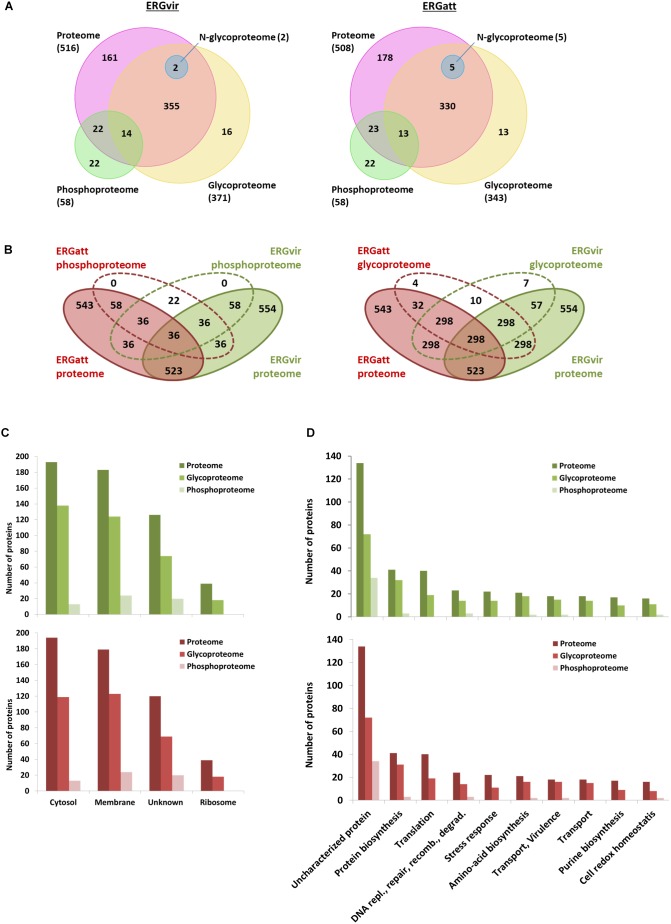
Characterization of ERGvir and ERGatt proteomes. **(A)** Number of identified proteins from proteome analysis (pink), phosphoproteome (green), glycoproteome (yellow), and *N*-glycoproteome (blue) enrichments for the ERGvir and ERGatt variants. **(B)** Overlap between proteins in the global proteome, phosphoproteome, and glycoproteome of ERGvir (green) and ERGatt (red). **(C,D)** Number of proteins identified in ERGvir (green) and ERGatt (red) in the global proteomes, phosphoproteomes, and glycoproteomes that were localized in the indicated four major cellular components **(C)** and that were involved in Top10 biological processes **(D)**, based on their GO.

This strategy led to identify with high confidence (probability ≥ 0.75) a total of 92 phosphopeptides, representing 58 phosphoproteins common to both ERGvir and ERGatt variants (Figure 2A,B and Supplementary Dataset S1). Among the identified phosphoproteins, 22 proteins were detected only in the phosphorylation data alone (Figure 2A,B and Supplementary Datasets S1, S2), indicating that phosphoproteins enrichment allowed detecting proteins that are typically of low abundance (Park et al., 2015). Phosphoproteins correspond to approx. 11% of the proteins identified in the global proteome (*n* = 58/554 for ERGvir and *n* = 58/543 for ERGatt) (Figure 2A,B and Supplementary Dataset S2).

Gene Ontology term analysis revealed that for both ERGvir and ERGatt, 41% of the identified phosphoproteins are membrane-associated (*n* = 24), 36% have unknown cellular localization (*n* = 21), and 22% are cytosolic (*n* = 13); no ribosomal phosphoproteins were detected (Figure 2C). In the Top10 of the “biological process” GO terms, 59% of the identified phosphoproteins were found to have unknown function (*n* = 34/58); annotated phosphoproteins span protein biosynthesis (*n* = 3), DNA replication/repair/recombination/degradation (*n* = 3), amino acid biosynthesis (n = 2), transport/virulence (*n* = 2), and cell redox homeostasis (*n* = 2) (Figure 2D and Supplementary Dataset S2). In both ERG variants, the two phosphoproteins involved in virulence (proteins ERGA_CDS_03830 and ERGA_CDS_04060) were homologous of AnkA and AnkB virulence-associated proteins in *A. phagocytophilum*, respectively. We also found the kinase pyruvate phosphate dikinase (Ppdk) (which participated in pyruvate metabolic process) and the phosphatase phosphatidylglycerophosphatase A (PgpA) (involved in lipid metabolism).

**Figure 3:**
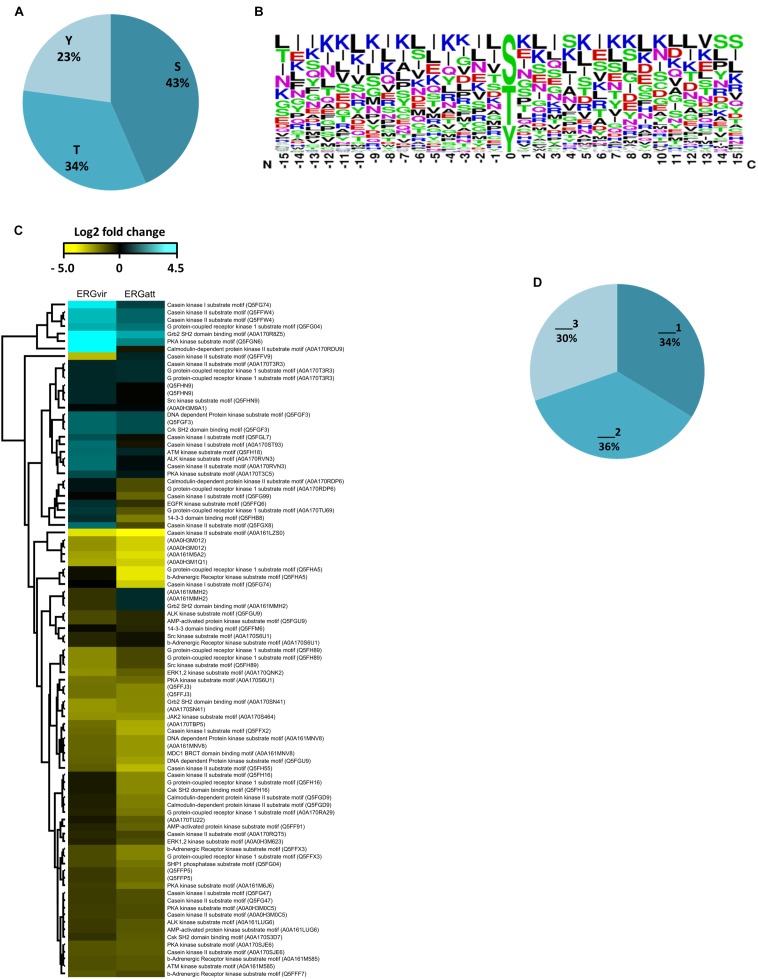
Amino-acid sequence features of localized phosphorylation sites in ERG variants. **(A)** Percentages of serine (S), threonine (T), and tyrosine (Y) phosphorylation sites identified. **(B)** Enriched phosphorylation motifs of bacterial phosphoproteomes obtained by Sequence logos analysis in Perseus software. **(C)** Heat map visualizing relative abundance of sequence motifs matching the indicated kinases from phosphopeptides identified (row color bar highlight proteins differentially expressed: blue, for increased abundance and yellow, for lower abundance). **(D)** Percentages of phosphopeptides single (_1), double (_2), and triply phosphorylated (_3) (“multiplicity”).

We also investigated the relative abundance of phosphorylation sites at serine, threonine, and tyrosine residues. We found that serines (S) account for 43%, threonines (T) for 34%, and tyrosines (Y) for 23% of all phosphorylation sites (Figure 3A).

As the amino acids surrounding the phosphorylated residues are important in determining the binding of a kinase to the protein sequence (of bacterial or host origin), we analyzed the substrate-binding motifs in the ERG-phosphorylated peptides (Figure 3B,C and Supplementary Dataset S1). Besides 18 phosphopeptides with unknown substrate motifs, we observed that motifs matching those recognized by Casein Kinase II (CKII), G protein-coupled receptor kinase 1 (GRK), CKI, and PKA kinases (all being S/T kinases) (Pinna and Ruzzene, 1996) are the most abundant one (Figure 3C and Supplementary Dataset S1). None of these kinases were detected in ER proteome (Supplementary Dataset S3).

We also found a high proportion of multiple phosphorylation sites (2 and 3), in up to 66% of the identified phosphoproteins for both variants (Figure 3D and Supplementary Dataset S1).

### Differentially Expressed Phosphoproteins

Label-free quantification of phosphoproteins was used to identify differential phosphorylation events in virulent and attenuated ER variants.

**Figure 4:**
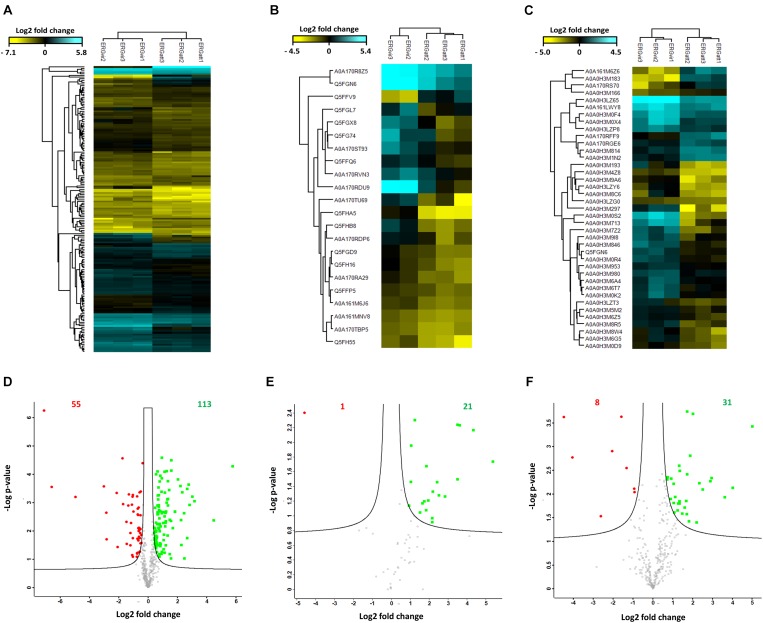
Differential expression of proteins in the proteomes of ERGvir and ERGatt. **(A–C)** Unsupervised hierarchical clustering using Euclidian distance of the proteome **(A)**, the phosphoproteome **(B)**, and the glycoproteome **(C)** data. Row color bar highlight proteins differentially expressed: blue, for increased abundance and yellow, for lowered abundance. **(D–F)** The volcano plots representing the quantitative analyses of the proteome **(D)**, the phosphoproteome **(E)**, and the glycoproteome **(F)** data. Significant changes in proteins, phosphoproteins, and glycoproteins in ERG were identified through a permutation-based FDR *t*-test (FDR = 0.05; S0 = 0.2). All results are based on at least two of three measurements per variant. The significant up-regulated proteins in ERGvir are colored in green and those in ERGatt, colored in red.

The heat maps in Figure 4A,B display proteins and phosphoproteins (respectively) that were quantified in at least two out of three biological replicates per variant and that passed an ANOVA-based multisample test for statistically significant up- or down-regulation, with a FDR of 5%. They allow visualizing the experimental specificity between variants and reproducibility within biological replicates: column-wise clustering of replicate measurements demonstrated that inter-biological variation was greater than intra-experimental variation. Proteins are color-coded according to their MS signal intensities, which is a relative measure for protein abundance (blue for increased abundance and yellow for lowered abundance). The expression pattern of identified proteins and phosphoproteins varies across the variants reflecting the phenotypic differences of the ERG variants.

Quantitative analyses of ERG proteomes revealed that more proteins are overexpressed in ERGvir (*n* = 113) compared to ERGatt (*n* = 55) (FDR < 0.05 and S0 = 0.2) (Figure 4D and Supplementary Dataset S3). In the phosphoproteome, we quantified 21 and 1 phosphoproteins differentially expressed (FDR < 0.05 and S0 = 0.2) in ERGvir and ERGatt, respectively (Figure 4E and Supplementary Dataset S2).

**Figure 5:**
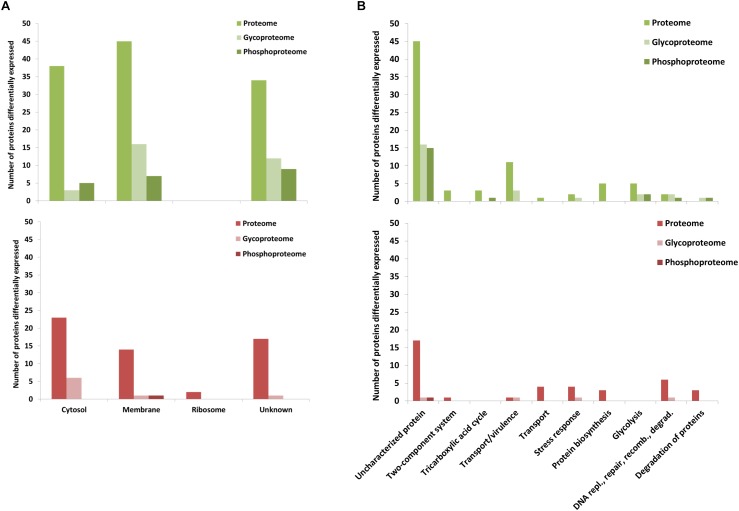
GO of the differentially expressed proteins of ERGvir and ERGatt proteomes, glycoproteomes, and phosphoproteomes. Number of proteins differentially expressed identified in the global proteomes, phosphoproteomes, and glycoproteomes that were localized in the indicated four major cellular components **(A)** and that were involved in Top10 biological processes **(B)**, based on their GO.

Gene Ontology terms analysis of the unique phosphoprotein overexpressed in ERGatt revealed that it is a membrane protein with unknown function (Q5FFV9) (Supplementary Dataset S2). The 21 phosphoproteins overexpressed in ERGvir have unknown cellular localization (*n* = 10), are located in the membrane (*n* = 6) or in the cytosol (*n* = 5) (Figure 5A and Supplementary Dataset S2). In the Top10 Go terms analysis, most of the overexpressed phosphoproteins in ERGvir have unknown functions (*n* = 15); the “biological process” GO terms associated with others cover glycolysis (*n* = 2/21) and tricarboxylic acid cycle, DNA replication/repair/recombination, and degradation of proteins (*n* = 3/21) (Figure 5B). Among the most upregulated phosphoproteins are ERGA_CDS_00630, ERGA_CDS_02510, and ERGA_CDS_04860 [all overexpressed in ERGvir with fold change (FC) > log 2, Supplementary Dataset S2]. All are uncharacterized proteins.

### Glycoproteome Characterization

The workflow for the enrichment of glycoproteins is illustrated in Figure 1B. ERGvir and ERGatt biological triplicates were harvested at host cell lysis and EB were purified by a multistep centrifugation process. After ERG lysis (Step 1), proteins extracts were either oxidized or not with sodium meta-periodate (Step 2) and incubated with pre-prepared ULH resin (Step 3). Glycoproteins were then enriched and two peptide fractions derived from an on-bead tryptic digestion (tryptic peptides of glycoproteins, TPG fractions) and a second fraction consisting of PNGase F-released peptides (*N*-glycopeptides, NGP fraction) (Step 4) were analyzed by LC–MS/MS for each biological sample (Step 5). Peptide identifications and protein expression analysis were done with MaxQuant followed by Perseus analysis (Step 6).

Combined, the analysis of TGP and NPG fractions resulted in the identification of 371 and 343 annotated glycoproteins detected in ERGvir and ERGatt datasets, including 64 and 36 variant-specific glycoproteins in ERGvir and ERGatt, respectively (Figure 2A,B and Supplementary Dataset S4). This corresponds to 67% (*n* = 371/554) in ERGvir and 63% (*n* = 343/543) in ERGatt of the total proteome. Among the TPG fraction, 16 and 13 proteins were detected only in the glycosylation dataset alone for ERGvir and ERGatt, respectively (Figure 2A and Supplementary Dataset S4). In the NPG fractions, only five proteins were detected confidently, three being specific to ERGatt and two shared by both variants (Figure 2A and Supplementary Dataset S4).

Gene Ontology term analysis of the identified proteins indicated overall similar cellular component and biological processes between ERGvir and ERGatt (Figure 2C,D). Subcellular compartment analysis revealed that the identified proteins are mainly located in the cytosol and in the membrane (Figure 2C). In the Top10 “biological process” Go term, besides the high number of uncharacterized glycoproteins (approx. 20%) in both variants, we observed that the identified glycoproteins are involved in several key biological processes such as protein and amino acid biosynthesis and also virulence (such as VirB2, VirB4, VirB6, VirB8, VirB9, VirB10, VirD4, AnkB AnkC, ApxR, Ats-1, and Bax-1-related protein). Many ribosomal proteins (*n* = 18) were detected in the glycoprotein fraction (Supplementary Dataset S4). In the TPG fraction, we also found 23 proteins that were detected in the ERGvir and ERGatt phosphoproteomes: ERGA_CDS_00630, ERGA_CDS_02360, ERGA_CDS_02470, ERGA_CDS_03680, ERGA_CDS_04060, ERGA_CDS_04760, ERGA_CDS_05650, ERGA_CDS_05790, ERGA_CDS_06390, ERGA_CDS_07690, ERGA_CDS_08310, Map 1–3, Gap, GpmI, GrxC2, HflK, PccB, PheS, PpdK, Thio, Tig, TktA, and Tld (Supplementary Dataset S2).

The identified *N*-glycoproteins (with the consensus sequence N–X–S/T, X = !P) present in both variants include VirB6 (involved in virulence) and the chaperone GroES. Interestingly, three additional *N*-glycoproteins were exclusively identified in ERGatt: Ptr1 (involved in co-factor biosynthesis) and two membrane-associated proteins, ERGA_CDS_05140 and ERGA_CDS_02770 (Supplementary Dataset S5).

### Differentially Expressed Glycoproteins

For label-free quantitative analysis, the proteomes of enriched glycoproteins of ERGvir and ERGatt were compared. The heat map in Figure 4C displays glycoproteins that were quantified in the three biological replicates per variant and that passed an ANOVA-based multisample test for statistically significant up- or down-regulation, with a FDR of 5%. Figure 4C also shows that inter-biological variation was greater than intra-experimental variation.

In the core glycoproteome, we quantified 31 and 8 proteins with significant changes (FDR < 0.05 and S0 = 0.2) in ERGvir and ERGatt, respectively (Figure 4F and Supplementary Dataset S4).

Subcellular compartment analysis revealed that up-regulated glycoproteins in ERGvir are mainly located in the membrane (52%, *n* = 16/31) or of unknown location (39%, *n* = 12/31), while in ERGatt, the differentially expressed proteins are mainly located in the cytosol (*n* = 6) (Figure 5A). Most glycoproteins in ERGvir are of unknown function (*n* = 16) compared to ERGatt (*n* = 1) (Figure 5B and Supplementary Dataset S4). The Top10 “biological process” GO terms associated with ERGvir and ERGatt glycoproteomes span Transport/Virulence, DNA replication, repair, recombination and degradation, and Stress response for both ERGvir and ERGatt, whereas Glycolysis and Degradation of proteins are only evidenced for ERGvir (Figure 5B). In ERGvir, the five most upregulated glycoproteins (FC > log2) are proteins with unknown function (ERGA_CDS_03680, ERGA_CDS_08310, ERGA_CDS_03410) and with exonuclease function (PolA, Rnd) (Supplementary Dataset S4). Proteins associated with virulence in other *Anaplasmatacae* such as Ats-1, ApxR, VirB4, and VirB8 are also found to be overexpressed in ERGvir (Supplementary Dataset S4). The glycoproteins NtrX (involved in transcription regulation), ERGA_CDS_04560 (uncharacterized protein), VirB2 (virulence-associated protein), ThiC (Co-factor biosynthesis), and FabI (Fatty acid biosynthesis) are overexpressed in ERGatt (FC > log2) (Supplementary Dataset S4).

### Identification of*O*-GlcNAcylated Proteins

**Figure 6:**
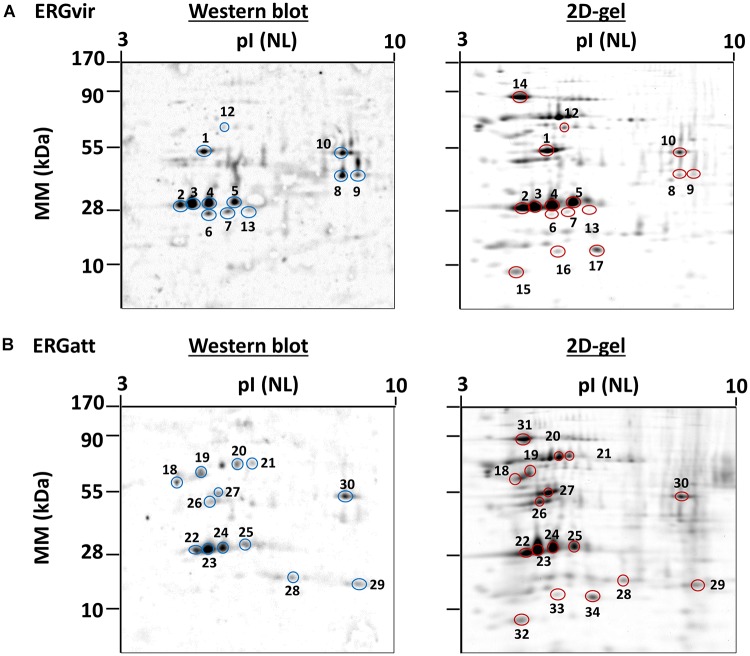
Detection of *O*-GlcNAcylated proteins in ERGvir **(A)** and ERGatt **(B)** using western blotting. 2D gels were used for western blotting (left) to detect specifically *O*-GlcNAcylated proteins or were stained with Coomassie (right) to detect total proteins. Protein spots identified as *O*-GlcNAcylated proteins (blue circles, in western blot) were excised from a corresponding 2D gels (yellow circles). Spots 14, 15, 16, and 17 (in ERGvir) and 31, 32, 33, and 34 (ERGatt) were only detected in the 2D gel and were used as control (for non-*O*-GlcNAcylated proteins). Each gel and western blot is representative of ERGvir and ERGatt variants. The protein identification for each spot is provided in Supplementary Dataset S6.

ERGvir and ERGatt protein extracts were separated by 2DE and stained with SYPRO Ruby or transferred to a nitrocellulose membrane and incubated with the monoclonal antibody CTD 110.6 (Figure 6A,B). Protein identification by MS revealed that the most prominent spots corresponding to Map-1 protein (spots 2, 3, 4, 5, 22, 23, 24, 25; Figure 6 and Supplementary Dataset S6) are *O*-GlcNAcylated, in both variants. Other proteins such as elongation factor Tu (spot 1), probable conjugal transfer protein trbG (VirB9, spots 6 and 7), VirB11 protein (spot 8), the porin ERGA_CDS_05140 (spot 9), ATP-dependent Clp protease ATP-binding subunit ClpX (spots 10 and 11), and Map1-1 protein (spot 13) were also found to be *O*-GlcNAcylated in ERGvir (Figure 6A and Supplementary Dataset S6). In ERGatt, proteins such as 60 kDa chaperonin (spots 12 and 18), transcription termination/antitermination protein NusA (spot 19), cell division protein FtsZ (spots 20 and 21), elongation factor Tu (spot 27), 50S ribosomal protein L25 (spot 28), peptide deformylase (spot 29), and ATP-dependent Clp protease ATP-binding subunit ClpX (spot 30) were found to be *O*-GlcNAcylated (Figure 6A and Supplementary Dataset S6). All of these proteins were detected in the TPG fraction (Supplementary Dataset S4). These results suggest that most of the proteins detected in the TPG fraction could be, in fact, *O*-GlcNAcylated proteins (especially in the case of proteins that are simultaneously phospho- and glycosylated, as reported above). To validate this hypothesis, we also picked some spots that were present in ERGvir and ERGatt 2D gels, but not in the western blot (Spots 14, 15, 16, and 17 for ERGvir; Spots 31, 32, 33, and 34 for ERGatt; Figure 6). The mass spectrometry results indicated the presence of GroEL, DnaK, rplL, ERGA_CDS_08670 proteins; these were not detected in the TPG fraction (Supplementary Dataset S4) but in the proteome (Supplementary Dataset S3). For the GroEL protein, we noticed that although it was detected in the TPG fraction, using western blotting, it was detected as non-glycosylated in ERGvir (Figure 6A, spot 12) and *O*-glycosylated in ERGatt (Figure 6B, spot 18).

## Discussion

Bacteria use PTMs to modify their own proteins [for adaptability or cell cycle control (Grangeasse et al., 2015)], but also those from the host to manipulate and regulate host processes and hereby promote infection (Salomon and Orth, 2013). Herein, we used an integrated proteomic approach to provide different layers of protein information (including protein abundance and PTMs) by analyzing the proteome, the S/T/Y phosphoproteome, and glycoproteome (with *O*- and *N*-modifications) to identify post-translationally modified proteins that could modulate ER physiology and pathogenesis.

### *Ehrlichia ruminantium* Phosphoproteomics

A number of global phosphoproteome studies have now been performed in bacteria, including *E. coli*, *Helicobacter pylori*, *Bacillus subtilis*, *Mycoplasma pneumoniae*, *Streptococcus pneumoniae*, *Klebsiella pneumoniae*, *Lactococcus lactis*, *Campylobacter jejuni*, *Pseudomonas* sp. (Whitmore and Lamont, 2012; Rioseras et al., 2018), and *Chlamydia* sp. (Claywell et al., 2016). Nevertheless, the whole phosphoproteome of *Rickettsiales*, and more specifically, *Ehrlichia* sp. has not been mapped so far. Herein, we present the first phosphoproteomes of two ER variants with different levels of virulence *in vivo* and different cell culture behavior *in vitro* (Marcelino et al., 2015). The annotated phosphoproteins are involved in biological processes such as DNA recombination and repair, cell metabolism, cell cycle and division, and also virulence and are located in the membrane. Many identified proteins remained uncharacterized, highlighting the need for further studies on their function. Most importantly, the identified phosphoproteins are present in both variants, indicating their relevance in the physiology of the extracellular and infectious form of the bacterium.

Protein phosphorylation plays a vital role in the regulation of many cellular processes (such as central carbon/protein/nucleotide metabolism, cell cycle, growth, apoptosis, etc.) but also in the monitoring and adapting to the dynamically changing environment in the host (Canova and Molle, 2014). To rapidly adapt to these changes, the bacteria can use environmental sensing systems such as two-component systems (TCSs) (Wang, 2012; Mijakovic et al., 2016). Several *Rickettsiales* (including ER) have the genes to code for three histidine kinases (homologs of, PleC, CckA, and NtrY) and their corresponding response regulators (homologs of PleD, CtrA, and NtrX) (Cheng et al., 2006). Herein, in ERGvir and ERGatt proteomes, we identified the response regulators NtrX, PleD, and CtrA, but only the sensor protein PleC was detected. No component of the TCS was found to be phosphorylated, neither in ERGvir nor ERGatt. Only the protein ERGA_CDS_06790 with a GGDEF domain (often associated to a regulatory domain, such as an oxygen sensing domain or a phosphorylation receiver) was found to be phosphorylated. Interestingly, these three pairs of TCSs were detected in *A. phagocytophilum* and *E. chaffeensis* (using immunofluorescence labeling and western blot analysis), but only during their intracellular development (Cheng et al., 2006); this strongly suggests that TCSs might be active mostly during the replicative phase of the bacteria.

Nowadays, it is well recognized that besides the TCS, bacteria can use, like eukaryotes, a network of protein phosphorylation cascades that require the synchronized action of a number of “eukaryote-like” serine/threonine and tyrosine kinases and their associated phosphatases (Canova and Molle, 2014). While kinases transfer a phosphate group from a nucleoside triphosphate (usually ATP) and covalently attach it to specific amino acids (such as S, T, and/or Y), phosphatases remove it. Kinases and phosphatases act as on/off switches and hereby modulate specific signal transduction pathways (Huse and Kuriyan, 2002). According to Frutos et al. (2006), ERG genome contains 22 kinases and 6 phosphatases-coding genes (Frutos et al., 2006) that could regulate phosphorylation events and, therefore, would play an important role in the control of biological processes such as proliferation, differentiation, and apoptosis. In this work, we identified 13 kinases and 5 phosphatases in the total proteome; among those, only pyruvate phosphate dikinase and phosphatidylglycerophosphatase A were found to be phosphorylated in ER EBs.

In bacteria, most phosphorylation sites are on Ser (70%) and Thr (20%), while Tyr phosphorylation sites account for less than 10% (Ge and Shan, 2011). A noteworthy feature of ER EBs phosphoproteome is the significantly high overall abundance (25%) of Tyr phosphorylation. To our knowledge, there is only one bacterial phosphoproteome with comparable Tyr phosphorylation percentage (*K. pneumoniae*, 25.8%) (Ge and Shan, 2011). Our results also show that approx. 30% of the identified Tyr-phosphorylated proteins are overexpressed in ERGvir. Several studies have established that Tyr phosphorylation is used as a key regulatory device of bacterial physiology, linked to virulence, stress response, and DNA metabolism (Grangeasse et al., 2015); this suggests that ERGvir pathogenesis and virulence could be related to increased levels of these Tyr-phosphorylated proteins. Tyr-phosphorylated proteins have been found in other *Anaplasmatacae*: the above mentioned, *E. chaffeensis* TRP47 (Wakeel et al., 2010) and also, the ankyrin repeat protein of *A. phagocytophilum*, AnkA. As for TRP47, AnkA was found to be Tyr-phosphorylated after delivery into the leukocyte cytoplasm, suggesting a tight interaction with the host (Ijdo et al., 2007; Lin et al., 2007). In our work, we observed that ERGA_CDS_03830 and ERGA_CDS_04060, the homologs of AnkA and AnkB virulence-associated proteins, respectively, were phosphorylated on Ser and Thr residues, respectively, instead of Tyr. Further studies are required to elucidate the impact of such difference during host cell infection.

Besides being specific to certain amino acids, kinases are also quite discriminating in their preference for particular peptides (Amano et al., 2015). In order to predict how well any given (previously untested) peptide will be phosphorylated by a given kinase, we analyzed the possible motifs of the identified phosphopeptides using Perseus. As mentioned above, the motifs matching those recognized by CKII, GRK, CKI, and PKA kinases (all being S/T kinases) are the most abundant ones in ER. None of these enzymes were detected in ER proteome [or are present in the genome (Frutos et al., 2006)], yet, they are present in the host (*Bos taurus*). This suggests that ER could use these phosphopeptides to interact with its host cell. As reviewed by Popa et al. (2016), some bacterial proteins do not exhibit an enzyme activity when expressed in bacterial systems, but rather require interaction with additional eukaryotic factors for full activation. This might also be the case for *ER*: indeed, for the ERG proteins to be targeted (and thus, phosphorylated) by the host, the bacterial protein should be exposed to the host environment during the infection, namely in the form of reticulated bodies. As we used the extracellular form of the bacterium as biological material, this could explain also the low number of phosphorylated proteins detected in this work.

Another interesting feature in the S/T/Y phosphoproteome of ERG variants is the presence of multiple phosphorylation sites. Phosphorylation at multiple sites within one domain or motif can be used to fine-tune delicate micro-regulations of mechanisms leading to pathogenesis, such as adhesion to and invasion of the host cells, stimulation and regulation of pathogenic functions, and weakening the host defense mechanisms (Ge and Shan, 2011). Our results revealed that ERG phosphopeptides have multiple phosphorylation sites: 35 out of the 92 identified phosphopeptides contain at least two phosphorylation sites. Multiple phosphorylation sites have been also reported in the characterization of the phosphoproteome of *H. pylori* and *L. lactis* (as reviewed by Ge and Shan, 2011). As both have small genome sizes, simple transcriptional machinery, and few sigma factors, it was suggested that these two bacteria could use this molecular strategy to perform delicate PTMs and hereby increase protein functionality and/or signal fine tuning (Ge and Shan, 2011). As ER has a comparable small genome, a similar regulation strategy *via* multiple phosphorylation sites could have been developed by this bacterium.

### *Ehrlichia ruminantium* Glycoproteomics

Glycoproteome characterization has been achieved in several bacteria, including the gastric pathogens *H. pylori* and *C. jejuni* (Longwell and Dube, 2013), but has never been explored in *Rickettsiales*. One of the most striking results obtained in this work is the high percentage (up to 67%) of glycoproteins detected in both variants. Our results show that ERGvir glycoproteome is composed of 371 glycoproteins while ERGatt has 343. These glycoproteins are involved in key biological processes such as protein, amino-acid and purine biosynthesis, translation, virulence, DNA repair, and replication. Among the proteins that constitute the core glycoproteome (307), only 2% are up-regulated in ERGatt while 10% are overexpressed in ERGvir. These include virulence-associated proteins such as VirB10, VirB2, VirB4, VirB6, VirB8, VirB9, VirD4, AnkB, AnkC, ApxR, Ats-1, and Bax-1-related protein. Protein glycosylation in bacteria has shown not to be vital for growth in laboratory conditions, yet it is essential in pathogenic bacteria (Poole et al., 2018) and this could explain why ERGvir has more specific proteins and higher expression levels of glycosylated proteins compared to ERGatt. Unfortunately, many proteins of potential interest with differential expression levels are of unknown function, and deserve further investigation to determine their functional role in the bacterial virulence.

Protein glycosylation is the process by which a carbohydrate is covalently attached to a protein on an asparagine (Asp, N) to form an N-linked glycan, or to serine (Ser, S) and threonine (Thr, T) residues to form an *O*-linked glycan (Valguarnera et al., 2016). In bacteria, *O*-glycosylation appears as a widespread phenomenon, while *N*-glycosylation of proteins seems to be more restricted (Dell et al., 2010), and our results demonstrated that it is the case also for ER. Indeed, while only five *N*-glycoproteins were identified in ERG variants, we detected at least 12 *O*-GlcNAcylated proteins per variant using 2D western blotting.

Glycans serve a variety of structural and functional roles in membrane and secreted proteins. For instance, they may contribute to modulate immune responses and to impact processes such as adhesion or invasion (Valguarnera et al., 2016; Stadlmann et al., 2017; Veillon et al., 2017). In ERG, approx. 35% of the identified glycoproteins are membrane-associated. Of particular interest are the proteins from the Map-1-family and the porin ERGA_CDS_05140 (“porin 5140”). These outer membrane proteins are the most abundant proteins in the ERG and present numerous proteoforms: 36 for Map-1 and 27 for porin 5140 (Marcelino et al., 2015). Map-1 is known to induce a strong immune response in the host, but its precise role in protective immunity against heartwater is not fully understood. Using 2D western blot, four different spots corresponding to Map-1 were detected in each variant and were positively identified as *O*-GlcNAcylated proteins. Different spots for the same protein might correspond to slight differences in glycan composition, that could affect Map-1 recognition by the host receptor, leading to a reduced (or impaired) inflammatory response *in vivo*. This strongly suggest that Map-1 protein could be used to trick the host immune system, as suggested elsewhere (Marcelino et al., 2015). Further studies regarding the characterization of the glycan associated to Map-1 should be performed to elucidate this hypothesis. Porin_5140 was found to be glycosylated in both ERGvir and ERGatt; still, in ERGatt, glycan is linked to Asparagine and therefore is *N*-glycosylated while in the virulent variant porin 5140 was found to be *O*-GlcNAcylated. The biological implication of this difference deserves further studies. As mentioned above, glycosylation has been detected on *Ehrlichia* and *Anaplasma* outer membrane proteins and TRPs. For instance, *E. chaffeensis* (TRP120 and TRP156) and *E. canis* (p140) proteins (McBride et al., 2000) were found to be glycosylated. *A. marginale* major surface protein 1 (MSP1) and 2 (MSP2) are glycosylated adhesins, involved in the attachment to vector and/or host cells, and could mimic lectins (De la Fuente et al., 2001; Garcia-Garcia et al., 2004; Dinglasan and Jacobs-Lorena, 2005). Our results suggest that ER could have developed comparable strategies (mimicry of lectins) to attach to the host cell target.

*Anaplasmatacae* utilize the bacterial Type IV secretion system (T4SS) to subvert the host innate immune system and exploit the host cell. The T4SS system is a multi-component membrane-spanning transporter machinery that translocates DNA to or from bacteria, and proteins or nucleoprotein complexes to the eukaryotic target cells (Alvarez-Martinez and Christie, 2009). Here, several “building blocks” of the ER T4SS such as VirB2 (homolog VirB2-8), VirB4, VirB6, VirB8, VirB9, and VirD4 protein were detected in ER glycoproteome. In the literature, the VirB10 protein was found to be *N*-glycosylated in *C. jejuni* (Nothaft and Szymanski, 2013); to our knowledge, no further information regarding the glycosylation of the T4SS components was found. Manual reannotation of proteins led us to identify ER bacterial effectors homologous to *A. phagocytophilum* proteins in the TPG fraction such as T4SS-secreted proteins AnkB, AnkC, Ats-1, and AprX proteins. We also identified a Bax inhibitor (BI)-1 like protein in ER glycoproteome. Ats-1- and BI-1-like protein are known to interfere with host cell apoptosis, and ApxR- and Ank-related proteins can regulate gene expression (Wang et al., 2007). These proteins could be used by ER to avoid death of the host cell during the infection process and to subvert the cell metabolism to its benefit and growth.

Protein glycosylation relies on glycosyltransferases activity to catalyze the transfer of an activated donor sugar to an appropriate acceptor, typically another sugar, lipid, protein, or small molecule. Despite all the glycoproteins detected in this work, according to bioinformatics analysis of *Ehrlichia* genomes, there is no evidence for the presence of conserved glycosyltransferases (Cantarel et al., 2009). Still, the possibility of the existence of a novel glycosyltransferase in the genome of ER should not be ruled out (Cheng et al., 2006). Members of the *Anaplasmataceae* family are enveloped with a rippled thin outer membrane which lacks thickening of the inner or outer leaflet and shows no sign of a peptidoglycan layer or lipopolysaccharide (LPS) (as reviewed in Lin and Rikihisa, 2003). This again emphasizes the poor ability of *Ehrlichia* species to produce glycans. Nevertheless, both the host (*B. taurus*) (Cantarel et al., 2009) and the vector (*Amblyomma* ticks) (Natala et al., 2013) have glycosyltransferases (GT19, 28, and 30). This result again emphasizes the intimate relation between *Ehrlichia* and its host (and/or vector) as the bacterium could take advantage of the host and/or vector PTMs machinery for its own proteins modification.

### PTMs Crosstalk in *Ehrlichia ruminantium*

In eukaryotes, the reversible modification of nuclear and cytoplasmic proteins with β-*O*-GlcNAc is important for transcriptional regulation, signaling, and protein–protein interactions, as mentioned above. Additionally, multiple PTMs have been documented to exist on the same protein, revealing an additional level of complexity (named “PTMs crosstalk”) (Hao et al., 2011; Leney et al., 2017). Due to the biosynthetic complexity of *O*-glycosylation, and contrary to *N*-glycoprotein detection, there are no consensus sequence motifs that can be used to predict and identify *O*-glycoproteins. Herein, based on the results obtained with immunodetection, we hypothesized that most of the proteins identified in the TPG are *O*-GlcNAcylated. Moreover, we also detected 23 proteins being both phosphorylated and glycosylated. This feature could explain why few phosphorylated proteins were detected in ERG, in particular ribosomal protein. Indeed, sites of *O*-GlcNAcylation are often found at or in close proximity to protein phosphorylation sites, and the intricate interplay (“PTMs crosstalk”) between both modifications is known to modulate many important processes in cells. Our results suggest that ER EBs use *O*-GlcNAcylation and phosphorylation as a major cellular signaling mechanism which could be primed for metabolism upon entry into the host cell.

## Conclusion and Prospects

This work strengthens previous reports on the existence of post-translationally modified proteins by glycosylation and phosphorylation in *Rickettsiales.* It also confirms that *Ehrlichia* uses these PTMs to generate multiple expressed protein forms and exploit them as regulatory devices to adapt itself to the different hostile environments encountered during its complex life cycle (mammalian and tick cells).

Most bacterial PTMs are dynamic and reversible. As such, we believe that the phosphoproteome presented herein is likely to be underrepresented, as phosphoproteomes are very dynamic. For instance, we speculate that *Ehrlichia* phosphoproteome would vary between early, middle, and late bacterial growth stages to accomplish the physiological needs of each developmental stage, and could represent a new way for selecting key molecular elements for the bacterium survival. We suggest that, while extracellular non-metabolically active EBs have reduced amount of phosphorylated proteins, these could be phosphorylated by interchange with *O*-GlcNAcylated within the host cell. More information about *Ehrlichia* phosphoproteome and the correlation between bacterial phosphorylation and potential pathogenicity will surely contribute to develop protein phosphorylation-targeted prodrugs for the control of bacterial infections.

The precise functions of bacterial glycoproteins in bacterial survival and adaptation and within host–pathogen interaction are just starting to arise. Understanding these roles represents new opportunities in the prevention of bacterial infections; this is of key importance especially when antibiotic resistance continues to increase. Indeed, bacterial glycoproteins represent attractive targets for new antibacterial treatments, as they are frequently linked to pathogenesis and contain distinctive glycans^2^ that are absent in mammalian hosts. The emerging studies of bacteria glycoprotein pathways enable setting the stage for the development of glycosylation-based therapeutic strategies, such as glycan-based vaccines to treat or prevent bacterial infection, especially to circumvent the use of antibiotics. Furthermore, as glycosyltransferases have not been identified in *Ehrlichia* sp. genomes, additional studies should be performed to define the mass of these glycoproteins; this would contribute to understand the extent and nature of the glycans (composition, structure, and attachment sites) on both the (native or recombinant) glycosylated proteins.

Globally, the data set reported herein represents groundwork and provides a useful resource for further hypothesis-driven studies of *Ehrlichia*-specific variations in phosphorylation and especially glycosylation-mediated regulation of proteins. It also opens a window of opportunity to identify new cellular targets for regulatory events.

## Author Contributions

IM and FC contributed to the conception or design of the work. IM, NC-C, PH performed the data collection. IM, NC-C, PH, FL, YR, FC, and NV executed data analysis and interpretation. IM drafted the article. All the authors performed a critical revision of the article and provided final approval of the version to be published.

## Conflict of Interest Statement

The authors declare that the research was conducted in the absence of any commercial or financial relationships that could be construed as a potential conflict of interest.

## Footnotes

1 http://proteomecentral.proteomexchange.org

2 http://csdb.glycoscience.ru/bacterial/

## Abbreviations

DC: dense core
2DE: bidimensional electrophoresis
EBs: elementary bodies
ER: *Ehrlichia ruminantium*
ERGatt: *Ehrlichia ruminantium* Gardel attenuated strain
ERGvir: *Ehrlichia ruminantium* Gardel virulent strain
FASP: filter-aided sample preparation
Hpi: hours post-infection
LFQ: label-free quantification
nanoLC: nano-scale liquid chromatography
nLC–MS/MS: nano-scale liquid chromatography–tandem mass spectrometry
PTM: post-translational modification
RBs: reticulate bodies
RC: reticulate core
RT: room temperature
S/T/Y: serine/threonine/tyrosine.
